# Leave It to the Prose: A Case of POEMS Syndrome

**DOI:** 10.7759/cureus.18664

**Published:** 2021-10-11

**Authors:** Samira Samant, Adrian Umpierrez De Reguerro

**Affiliations:** 1 Internal Medicine, Froedtert & the Medical College of Wisconsin, Milwaukee, USA

**Keywords:** polyneuropathy, endocrinopathy, plasma cell dyscrasia, monoclonal gammopathy, poems syndrome

## Abstract

POEMS syndrome is a rare paraneoplastic syndrome named for its constellation of polyneuropathy, organomegaly, endocrinopathy, monoclonal protein, and skin changes. As a proliferative plasma cell disorder, it has a multisystem presentation and chronic cytokine overproduction. It often presents in the fifth or sixth decade of life, mostly in males. Presentations vary widely, making it extremely difficult to diagnose. A chronic progressive distal, sensorimotor polyneuropathy in conjunction with a monoclonal plasma cell dyscrasia suggests POEMS.

A sixty-one-year-old man with a significant past medical history of bilateral lower extremity weakness and a cerebral meningioma presented with anorexia, intermittent dysphagia, and early satiety with intermittent vomiting and diarrhea over the past three months with a 20-pound weight loss. A CT of the abdomen and pelvis was obtained due to vomiting, showing expansile lesions of the left ischium, acetabulum, and ilium, with small-volume ascites and splenomegaly. Because of these findings and the noted bilateral lower extremity neuropathic weakness, we obtained further testing to corroborate suspicions of a plasma cell disorder. Labs were significant for IgG lambda with a monoclonal spike (M spike), low cortisol, low testosterone, and elevated thyroid-stimulating hormone (TSH). Protein electrophoresis was positive for bi-clonal lambda, and his vascular endothelial growth factor (VEGF) levels were elevated to 377. Bone marrow core biopsy indicated malignancy. Given his polyneuropathy, organomegaly, endocrinopathy, IgG lambda, skin changes, and extravascular fluid overload, POEMS syndrome was diagnosed. He was initiated on lenalidomide and dexamethasone and eventually received a stem cell autologous bone marrow transplant.

Given his non-specific presentation, and the confounding factor of a known meningioma causing hypopituitarism, diagnosing POEMS required extensive workup. A contrast CT demonstrating bone lesions associated with myeloma-type disease was crucial, pairing his endocrinopathy and neuropathy with a plasma cell dyscrasia. Although POEMS is exceedingly rare, accurate diagnosis is vital, as treatment requires a multidisciplinary approach. While high-dose chemotherapy-conditioned autologous stem cell transplantation is the gold-standard treatment for POEMS syndrome, patients who are diagnosed in a late stage of the disease are not candidates, underlining the need for early identification of the disorder.

## Introduction

POEMS syndrome is a rare paraneoplastic syndrome that is named for its constellation of polyneuropathy, organomegaly, endocrinopathy, monoclonal protein, and skin changes. It is a proliferative plasma cell disorder, often lambda-restricted, and its classic multisystem presentation distinguishes it from other similar syndromes, such as monoclonal gammopathy of undetermined significance or immunoglobulin light chain amyloid neuropathy [1]. The etiology of POEMS remains unclear. Chronic overproduction of proinflammatory and other cytokines is characteristic, higher than that of typical multiple myeloma [2,3].

POEMS commonly presents in the fifth or sixth decade of life, with 63% of patients being of the male gender [1,4]. Presentations and multisystem involvement vary widely, making this syndrome extremely difficult to diagnose. However, a chronic progressive distal, sensorimotor polyneuropathy in conjunction with a monoclonal plasma cell dyscrasia suggests POEMS, and warrants further workup.

## Case presentation

A sixty-one-year-old man with a significant two-year past medical history of bilateral lower extremity weakness and a left parasellar meningioma presented with anorexia, intermittent dysphagia, and early satiety with intermittent nausea, vomiting, and diarrhea from which he had been suffering for the past three months. During this time, he experienced a 20-pound unintentional weight loss. He was not taking any medications known to cause peripheral neuropathy; additionally, he reported a 20 pack/year smoking history and denied alcohol or illicit substance use.

Prior to admission, in January 2016, he had undergone extensive workup for similar symptoms of abdominal pain, nausea, vomiting, and dysphagia. The resulting workup included a CT of the chest, abdomen, and pelvis that demonstrated a fatty infiltrative change in the liver and a kidney, ureter, and bladder X-ray that showed a nonobstructive abdominal gas pattern with no dilated bowel loops or masses identified. An esophagogastroduodenoscopy done in March 2016 demonstrated dysphagia likely secondary to underlying neurologic issues, along with a small hiatal hernia, and an MRI that June revealed an unchanged left middle cranial fossa mass consistent with meningioma.

Upon presentation in July 2016, our patient reported bilateral lower extremity pain present over the past six months, which had been attributed to distal axonal motor neuropathy by electromyogram and nerve conduction studies by his outpatient neurologist. He characterized his pain as alternating between throbbing or shooting up his legs, and that he had been started on pregabalin six months prior with some amelioration of his symptoms. Oxcarbazepine had been recently added to his medication, with some results. He also reported weakness in both his feet since receiving radiation therapy for meningioma two years prior, requiring the use of a walker to ambulate.

In the emergency department, his creatinine was elevated to 1.31 mg/dL from a baseline of 0.9 mg/dL, and troponins were elevated threefold with a normal EKG. His physical exam was significant for splenomegaly and small-volume perihepatic ascites contributing to abdominal distension. He was admitted for acute kidney injury.

**Figure 1:**
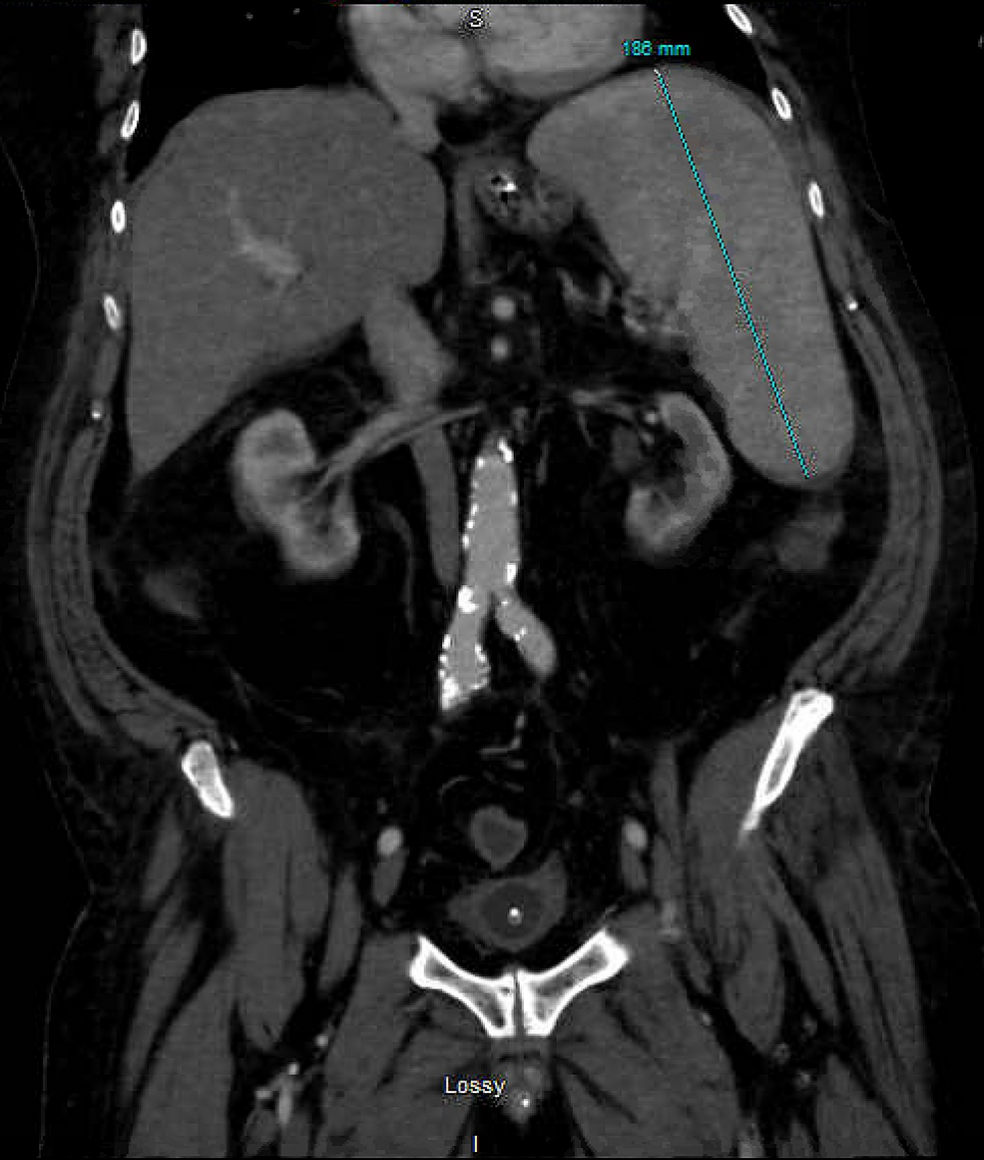
Moderate splenomegaly of 18.6 cm

**Figure 2:**
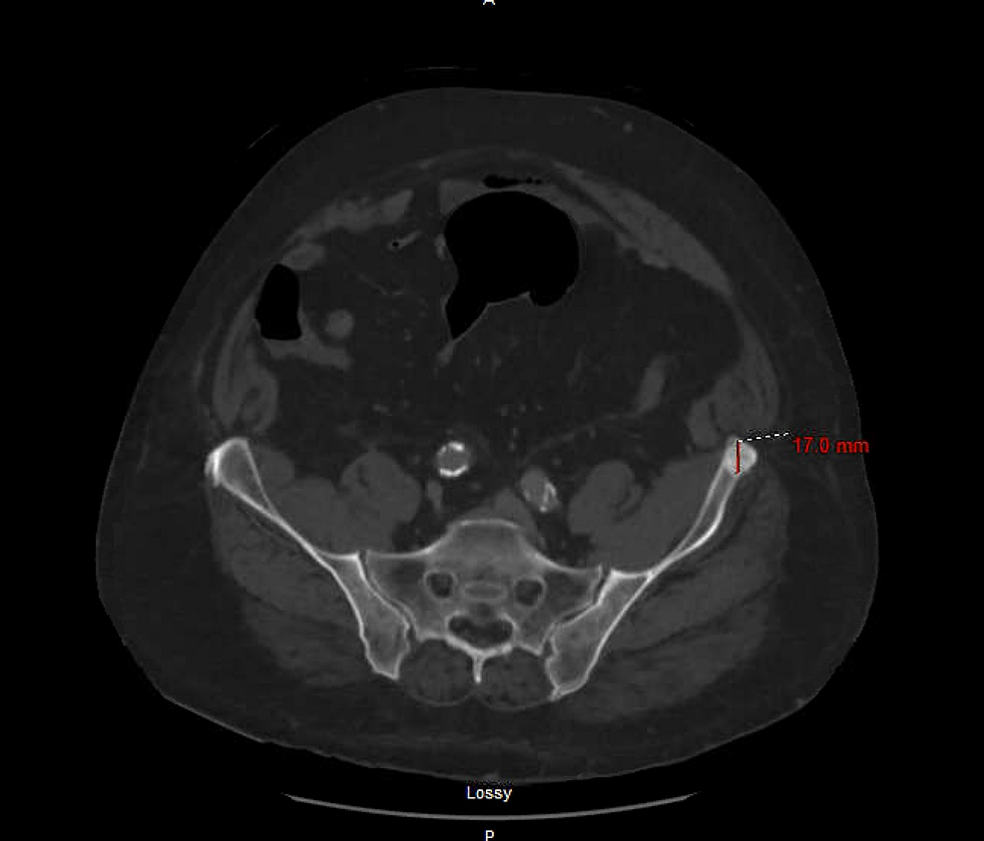
Sclerotic focus of 1.7 cm in the left ilium

A CT of his abdomen and pelvis was obtained, revealing small-volume peri-hepatic ascites, splenomegaly (Figure 1), and incidentally-noted expansile lesions of the left ischium, acetabulum, and ilium (Figure 2).

Due to these findings and noted bilateral lower extremity neuropathic weakness, we obtained further testing to corroborate our suspicions of a plasma cell disorder. Laboratory data were significant for monoclonal gammopathy (bi-clonal IgG lambda on serum protein electrophoresis), as well as marked endocrinopathy: adrenal insufficiency with a low AM cortisol of 3.6 ug/dL; hypothyroidism with a TSH of 3.76 uIU/mL and low free thyroxine (T4) of 0.45 ng/dL; and hypogonadism with low free testosterone of 0.3 pg/mL. His VEGF levels were elevated to 377 pg/mL. Bone marrow core biopsy confirmed our suspicions of a plasma cell dyscrasia; the core biopsy was 50% cellular, with plasma cells comprising 7% of the smear cellularity, and of which approximately half were noted to be immunophenotypically abnormal.

Given his polyneuropathy, organomegaly, endocrinopathy, IgG lambda, skin changes (leukonychia and rubor in his extremities), and extravascular fluid overload, he was diagnosed with POEMS syndrome. He was initiated on lenalidomide and dexamethasone following diagnosis, and eventually received an autologous stem cell bone marrow transplant.

## Discussion

Given the patient’s nonspecific presentation, in conjunction with the confounding factor of a known meningioma causing hypopituitarism, diagnosing POEMS required a high clinical suspicion and tailored workup. A contrast CT demonstrating bone lesions indicative of myeloma-type disease was crucial, pairing his endocrinopathy and neuropathy with a plasma cell dyscrasia.

Diagnosis of POEMS is made with the identification of mandatory criteria of polyneuropathy and a monoclonal plasma cell proliferative disorder, typically lambda-type light chain, both of which our patient presented with [5]. Additionally, one other major criterion (Castleman disease, sclerotic bone lesions, or VEGF elevation) and one minor criterion (organomegaly, extravascular volume overload, endocrinopathy, skin changes, papilledema, or thrombocytosis/polycythemia) are required [5,6]. While endocrine involvement is common in POEMS, hypogonadism is the most frequently encountered endocrinopathy in this condition, followed by hypothyroidism and diabetes mellitus; the prevalence of the latter two conditions in the general population precludes endocrinopathy from being a major criterion in diagnosis [7]. Neuropathy - specifically a peripheral, ascending, symmetric neuropathy affecting sensory and motor function - is frequently the primary presenting complaint in POEMS syndrome [6].

Prior to the use of autologous stem cell transplants and high-dose chemotherapy, median survival was about 14 years; 10-year survival rates have since risen to 79% for those patients diagnosed after 2003 [8,9]. Some clinical factors associated with increased overall mortality have been identified, including ages over 50, development of a pleural effusion, estimated glomerular filtration rate (eGFR) < 30 ml/min/1.73m^2^, and pulmonary hypertension [10]. Extravascular fluid overload, typically manifesting as effusions, edema, or ascites is also associated with decreased survival [8,10]. Thrombocytosis and bone marrow infiltration are associated with an increased risk for cerebrovascular accidents; moreover, the presence of thrombocytosis assists in differentiating POEMS from chronic inflammatory demyelinating polyneuropathy [10,11]. Lower VEGF levels on presentation have been correlated with more robust responses to therapy; VEGF is also a useful marker of therapeutic response, and a reduction in VEGF following treatment typically correlates well with prognosis [9,12].

The differential diagnosis for POEMS is incredibly broad, including multiple myeloma, solitary plasmacytoma of bone, monoclonal gammopathy of undetermined significance, Waldenstrom macroglobulinemia, amyloid light-chain (AL) amyloidosis, cryoglobulinemia, and chronic inflammatory demyelinating polyradiculoneuropathy [6,10,13]. As there are several conditions that present with plasma cell dyscrasia and polyneuropathy and which may be associated with osteosclerotic bone lesions, the diagnosis of POEMS is understandably complex.

Although POEMS is exceedingly rare, accurate diagnosis is vital, as treatment requires a multidisciplinary approach that includes hematology, neurology, neurophysiology, and physiotherapy [5,6,13]. The therapeutic goal is to ameliorate symptoms while addressing the underlying solitary plasmacytoma and reducing VEGF and other inflammatory cytokine levels [13]. High-dose chemotherapy-conditioned autologous stem cell transplantation is the gold-standard treatment for POEMS syndrome, especially if bone lesions are present [13-15]. However, autologous stem cell transplantation is not without significant risks, including engraftment syndrome, graft failure, or pancytopenia, and severe infection [5]. Patients who are diagnosed in a late stage of POEMS are often not candidates, underlining the need for early identification of the disorder despite the complexity of the diagnosis [13].

## Conclusions

Given the clinical variety and complexity of POEMS, the resulting diagnostic challenge it presents has led to this condition being under-recognized and under-diagnosed. In presenting our patient’s case, we hope to contribute to the growing body of literature concerning POEMS syndrome to further elucidate possible presenting symptoms and diagnostic paradigms. While further research is needed and ongoing to evaluate the therapeutic options for POEMS syndrome, we hope that enhanced clinical clarity regarding this condition may prove useful in better understanding treatment options and outcomes.
